# A novel enterovirus species identified from severe diarrheal goats

**DOI:** 10.1371/journal.pone.0174600

**Published:** 2017-04-04

**Authors:** Mingyue Wang, Jia He, Haibing Lu, Yajing Liu, Yingrui Deng, Lisai Zhu, Changming Guo, Changchun Tu, Xinping Wang

**Affiliations:** 1 College of Veterinary Medicine, Jilin University, Changchun, Jilin, China; 2 Institute of Military Veterinary, Academy of Military Medical Sciences, Changchun, Jilin, China; 3 Key laboratory for Zoonosis, Ministry of Education, Changchun, Jilin, China; University of Hong Kong, HONG KONG

## Abstract

**Backgrounds:**

The *Enterovirus* genus of the family of *Picornaviridae* consists of 9 species of *Enteroviruses* and 3 species of *Rhinoviruses* based on the latest virus taxonomy. Those viruses contribute significantly to respiratory and digestive disorders in human and animals. Out of 9 *Enterovirus* species, *Enterovirus* E-G are closely related to diseases affecting on livestock industry. While enterovirus infection has been increasingly reported in cattle and swine, the enterovirus infections in small ruminants remain largely unknown.

**Methods:**

Virology, molecular and bioinformatics methods were employed to characterize a novel enterovirus CEV-JL14 from goats manifesting severe diarrhea with morbidity and mortality respectively up to 84% and 54% in China.

**Results:**

CEV-JL14 was defined and proposed as a new *Enterovirus* species L within the genus of *Enterovirus* of the family *Picornaviridae*. CEV-JL14 had a complete genome sequence of 7461 nucleotides with an ORF encoding 2172 amino acids, and shared 77.1% of genomic sequence identity with TB4-OEV, an ovine enterovirus. Comparison of 5’-UTR and structural genes of CEV-JL14 with known *Enterovirus* species revealed highly genetic variations among CEV-JL14 with known *Enterovirus* species. VP1 nucleotide sequence identities of CEV-14 were 51.8%-53.5% with those of *Enterovirus* E and F, 30.9%-65.3% with *Enterovirus* G, and 43.8–51. 5% with *Enterovirus* A-D, respectively. CEV-JL14 was proposed as a novel species within the genus of *Enterovirus* according to the current ICTV demarcation criteria of enteroviruses.

**Conclusions:**

CEV-JL14 clustered phylogenetically to neither *Enterovirus* E and F, nor to *Enterovirus* G. It was defined and proposed as novel species L within the genus of *Enterovirus*. This is the first report of caprine enterovirus in China, the first complete genomic sequence of a caprine enterovirus revealed, and the unveiling of significant genetic variations between ovine enterovirus and caprine enterovirus, thus broadening the current understanding of enteroviruses.

## Introduction

The genus *Enterovirus* within the family of *Picornaviridae* consists of 9 species (A, B, C, D, E, F, G, H and J) of *Enteroviruses* (EV) and 3 species (A, B, C) of *Rhinoviruses* based on the latest virus taxonomy[1]. Those viruses are the etiological agents contributing to neurological, respiratory and digestive disorders in human and animals[2–8]. Out of 9 *Enterovirus* species, *Enterovirus* E (EV-E), *Enterovirus* F (EV-F) and *Enterovirus* G (EV-G) are closely related to diseases affecting on the livestock industry, where EV-E and EV-F (formerly named bovine enterovirus A and B) are the causative agents of enterovirus infections in cattle manifesting clinical signs varying from respiratory diseases to enteritic, reproductive disease and infertility[4, 5, 7–11]. The EV-G (formerly named porcine enterovirus B) is the causative agents of swine enterovirus infections[6, 12, 13]. It has been demonstrated that EV-E and EV-F were usually isolated from the cattle manifesting symptoms of digestive and respiratory diseases, suggesting the potential pathogenicity of enterovirus related to those infections. EV-E and EV-F were also occasionally isolated or detected from cattle of subclinical infection or environment[14–17], which leads to the conclusion that EV-E and EV-F were not significant agents to the livestock industry. With increasing number of enteroviruses isolated from animals developing respiratory and digestive diseases, the pathogenicity of enteroviruses to animal has been drawn much attention[7, 8, 11, 13, 18]. While infection of enteroviruses and their sequences have been increasingly reported in cattle and swine recently, the enterovirus infections in small ruminants such as sheep or goat remain largely unknown. Recently, the first complete genome sequence of an ovine enterovirus (TB4-OEV) was reported in 2012[5], a partial 5’-UTR sequence of a caprine enterovirus in Japan and several BEV-like 5’-UTR sequences in Thailand were determined from diarrheal goats or unidentified goats[8, 19]. Although the phylogenetic analysis has clustered those sequences to clades of BEV-like based on the 5’-UTR sequences[19], such an analysis need to be reevaluated since the paucity of enough enterovirus sequences from petite ruminants including sheep and goats. It was reported that TB4-OEV strain is likely an interspecies recombinant between bovine enterovirus and porcine enterovirus based on the virus genome sequence analysis, where its 5’-UTR sequence has a high sequence identity with EV-E and EV-F, and the rest of its genome has a high sequence identity to EV-G[5]. Phylogenetic analysis grouped the TB4-OEV as a member of the species EV-G currently.

Classification for enterovirus species is usually based on virus genetic variability and molecular difference, in which the sequences of 5’-UTR or the capsid protein regions were normally used. It is generally accepted that picornavirus serotypes are molecularly defined by the diversity of the capsid proteins, while the enterovirus species were determined by the less diverse non-structural protein regions[20–23]. These criteria have been used successfully to classify bovine enteroviruses to EV-E, EV-F and porcine enterovirus to EV-G as well as other enterovirus classification. Previously, we defined an enterovirus HY12 isolate from cattle manifesting severe digestive and respiratory diseases as EV-E[7]. Here, we reported the isolation and characterization of the enterovirus isolate from goats showing severe diarrhea with high morbidity and mortality in China, and proposed that CEV-JL14, along with TB4-OEV, shall be grouped to a novel designated enterovirus species L (EV-L) within the genus of *Enterovirus*. To our knowledge, this is the first complete caprine enterovirus genome revealed, which is potentially associated with enteric disease characterized with severe watery diarrhea with high morbidity and mortality.

## Materials and methods

### Ethics statement

The diseased goats were sacrificed following a standard protocol approved specifically to this study by the animal ethics committee at Jilin University.

### Virus isolation

Fecal samples were collected from diarrheal goats. Spleen samples were collected from the dead or sacrificed goats and processed following the protocols approved by the animal ethics committee at Jilin University. Briefly, fecal samples were processed as described previously[7]. Spleen samples were processed in a dilution of 1:10 (W/V) with 10 mM phosphate buffered saline (PBS) (pH7.2) and homogenized. After centrifugation at 12,000 × g for 15 min at 4°C the supernatants were filtered with 0.45 nm filter and the flow-through was used to inoculate Vero cells with a confluency of 70–80%. The inoculums were discarded after incubation with cells for 2 h before the addition of Dulbecco's modified eagle's medium (DMEM) (Invitrogen) supplemented with 2% fetal bovine serum (Sigma), 2 μg/ml Gentamycin and 2 mM L-glutamine (Invitrogen). The cells were observed every 4–6 h, and cytopathic effects (CPE) were captured using Canon Digital Camera.

### Electron microscope observation

Fecal, spleen and the infected cell culture samples were processed for EM observation as previously described[7]. Briefly, fecal and spleen samples were processed in 1:10 (w/v) with 10 mM, pH7.2 PBS, and homogenized before centrifuging at 8000 × g for 20 min at 4°C. The infected cells were frozen and thawed for 3 times before centrifugation as described above. The supernatants were then incubated with 1% phosphotungstic acid, and the viruses were examined using electron microscope (JEM-2200FS/CR)(JEOL, Tokyo, Japan).

### TCID_50_ titration

TCID_50_ was determined as previously described[24]. Briefly, the viruses were processed in 10-fold serial dilutions and used to infect quadruplicate wells for each dilution. After 48–72 h post infection, the wells with cytopathic effects were counted and TCID_50_ was determined. Physicochemical properties of CEV-JL14 were characterized as described previously[7]. Viruses were treated with organic solvents (chloroform and ether), heated at 56°C, 65°C and 80°C for 1 h, or incubated with DMEM of pH 3.0 and pH5.0 before infecting the cells.

### RT-PCR amplification and determination of the complete genome sequences

Reverse transcriptase reaction was performed using SuperScript^™^ II Reverse Transcriptase (Invitrogen, Carlsbad, CA) following manufacturer’s instruction. PCR amplification was done using Taq DNA polymerase (New England Biolabs, Ipswich, MA) after the conditions being optimized. The primers used for determination of the potential pathogens and for amplification of the complete genomic sequences were listed in Table 1. The complete genome sequence was amplified by PCR using high fidelity PCR kit (Takara, Dalian, China). PCR products were either directly sequenced or cloned into pGEM-T vector (Tiangen, Beijing) before sequencing. Each product or recombinant was sequenced at least twice. The complete genome sequence was obtained by joining the nucleotide sequences of several overlapping PCR-amplified fragments using DNAstar Lasergene software. The nucleotide sequence served as a template for searching homologous sequences through GenBank (www.ncbi.nlm.nih.gov).

**Table 1:** Primers used for detecting potential pathogens and amplifying the complete genome sequences.

Fragment	primer sequence (5’-3’)	positions
BPV-S	GCGCCGCATAAATGTGTCTTGGTG	1326–1349
BPV-AS	TGTGGGGCTTTCCGCTTTATCTCA	1738–1715
FMDV-S	ACGGTGGAAAACTACGGGGGAGAG	40–63
FMDV-AS	CTGCGCCGTAGTTGAAGGAGGTTG	496–473
CEV-S	AGGATGATGATTGGCAGATTTTGT	372–395
CEV-AS	CATGTGGAAGTGTCTTTTGAGGAA	708–685
F1-S	TTTAAAACAGCCTGGGGGTTGTA	1–23
F1-AS	ATTAGCAGCATTCACGGCCCCG	546–525
F2-S	CGGGGCCGTGAATGCTGCTAAT	525–546
F2-AS	GGGGCTCGAATCCTCATCTGTGG	1826–1804
F3-S	CACCCGGCTCGTATCAGTTTCTTA	1780–1803
F3-AS	ATCCGGTGCCATCCTTTACA	2886–2867
F4-S	CACCAGCHCTDCAGGCGGCHGAAA	2590–2613
F4-AS	CCCCCACAGTCHCCAGGCTTACA	3667–3645
F5-S	GATCAAGCTCCGGGTCTATGC	3185–3205
F5-AS	CTGGGCTTCAAGGAGGGGTAA	4256–4236
F6-S	TAAGGGCCTAGATTGGGTTGGTGA	4124–4147
F6-AS	GTGGGCGTGCCTGGGAAGC	5525–5507
F7-S	TCTGCGCCGCTCTGAAA	5908–5924
F7-AS	AGCCCGCCCGACTGGAACTGA	7280–7260
F8-S	ACGCCCACCCAGCCGAACA	5518–5536
F8-AS	GCTGGACGCCTCAATCAACCTACT	6488–6465
3’-UTR-Out	TGCGTACGGGGATGATGTTATTG	6920–6940
3’-UTR-in	CTACACATGACACCCCCAGATAAG	7002–7025

BPV: bovine parvovirus (AF406967.1); FMDV: foot and mouth disease virus (FJ785290.1); CEV-S/CEV-AS and F1-S were designed based on HY12 strain (KF748290.1); the primers F1-A to 3’-UTR-in were designed based mainly on TB4-OEV (JQ277724.1); CEV: caprine enterovirus; S stands for sense; AS refers to antisense.

### Phylogenetic analysis

Alignment analysis of multiple sequences was performed using the Clustal W method[25]. The amino acid sequence of CEV-JL14 was deduced from the nucleotide sequence. Briefly, the complete nucleotide sequence of CEV-JL14, the polyprotein, the 5’-UTR nucleotide sequence, the deduced amino acids of VP1, P1, P2C and P3CD of CEV-JL14 were aligned with the corresponding regions of representative virus strains of EV-E, EV-F, EV-G and other enterovirus species available in the GenBank. The representative strains were listed in Table 2. Phylogenetic tree was initially generated by neighbor-joining methods[26], and further analysis was performed with a bootstrap value of 500.

**Table 2:** List of enterovirus strains used for phylogenetic analysis.

Name of strains	Sequence	Accession No	Country	Collection date	species	Host
BEV-3A	complete	AY508697	USA	2004	F	bovine
BEV-261	complete	NC_021220	USA	2014	F	bovine
BEV-BHM26	complete	HQ917060	CHINA	2012	F	bovine
BEV-BJ001	complete	HQ663846	CHINA	2014	F	bovine
BEV-PS 87	complete	AY508696	UK	2004	F	bovine
BEV-LC-R4	complete	DQ092769	USA:PA	2013	E	bovine
BEV-PA12-24791	complete	KC667561	USA	2013	E	bovine
BEV-PS 83	complete	DQ092793	USA	2013	E	bovine
BEV-HY12	complete	KF748290	CHINA	2014	E	bovine
BEV-SL305	complete	AF123433	Australia	1999	E	bovine
CEV-JL14	complete	KU297674	CHINA	2014	L	caprine
PEV10- LP 54	complete	AF363455	UK	2002	G	pig
PEV-B-KOR	complete	JQ818253	South Korea	2009	G	pig
PEV-K23-2008-HUN	complete	HQ702854	Hungary	2008	G	Sus scrofa
PEV-WBD-2011-HUN	complete	JN807387	Hungary	2011	G	Sus scrofa
TB4-OEV-2009-HUN	complete	JQ277724	Hungary	2009	?	Ovis aries
Goat enterovirus G1	Partial-5’UTR	AB857843	Japan	2014	L?	goat
Goat EV-Thailand-F27	Partial-5’UTR	KT992118	Thailand	2013	L?	goat
Goat EV-Thailand-F30	Partial-5’UTR	KT992119	Thailand	2013	L?	goat
Goat EV-Thailand-F32	Partial-5’UTR	KT992120	Thailand	2013	L?	goat
Goat EV-Thailand-F36	Partial-5’UTR	KT992121	Thailand	2013	L?	goat
Goat EV-Thailand-F40	Partial-5’UTR	KT992122	Thailand	2013	L?	goat
Goat EV-Thailand-F42	Partial-5’UTR	KT992123	Thailand	2013	L?	goat
CBV3-18219-02	complete	AY896763.1	Moldova	2002	B	human
EV11-18744-02	complete	AY896764.1	Moldova	2002	B	human
EV7-15936-01	complete	AY896765.1	Azerbaijan	2001	B	sewage
EV30-8477-98	complete	AY896767.1	Russia	1998	B	Human with meningitis
S0098b/CA16/2013/CHN	complete	KM402020.1	China	2013	A	Homo sapiens
S0102b/EV71/2013/CHN	complete	KM402021.1	China	2013	A	Human feces
Sev-nj1	complete	KT581587.1	China	2013	J	primate
1631	complete	NC_010415	USA	2007	J	Simian
EV-D68	complete	KX351830.1	USA	2014	D	Homo sapiens
Beijing-R0132	complete	KP240936.1	China	2014	D	Homo sapiens
US/KY/14-18953	complete	KM851231.1	USA	2014	D	Homo sapiens
Brunenders	complete	KP793687.1	German	1952	C	Homo sapiens
V2-Tol.1	complete	HQ738303.1	Madagascar	2005	C	Homo sapiens
V3-Tul.7	complete	HQ738302.1	Madagascar	2005	C	Homo sapiens

### RNA secondary structure prediction

The secondary structures of 5’-UTR for CEV-JL14 was predicted using minimal free energy as previously described[27], and compared with that of TB4-OEV, PEV10 LP54, HY12, BHM26, and EV71.

## Results

### Outbreak of disease with a high morbidity and mortality

In April, 2014, a disease occurred on a goat farm in Jilin province in Northeast China characterized by goats manifesting pyrexia, fever, cough at the onset. Three to five days later, the goats showed respiratory distress, dyspnea and severe diarrhea. No obvious improvement was observed after they were treated with antibiotics or sulfa drug. Majority of the sick goats succumbed to death within 7–14 days. The recovered goats showed a wasting and curved-body hair. During the outbreak, 64 out of 76 goats in a barn showed some obvious clinical signs, 35 out of 64 diseased goats were deceased. The morbidity rate is 84% (64/76) and the mortality is 54% (35/64).

### EM observation and virus isolation

**Fig 1:**
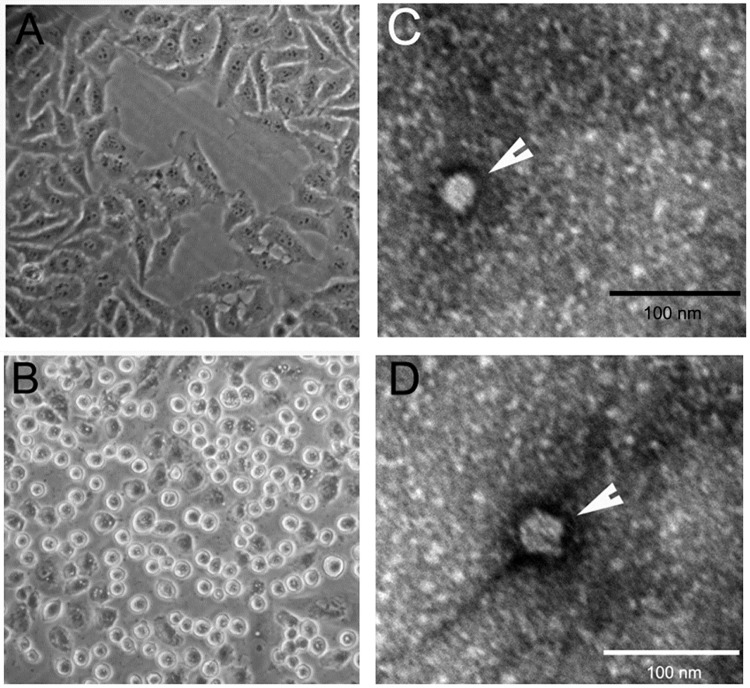
Microphotograph showing cytopathic effects and viruses in Vero cells inoculated with processed fecal samples and spleen samples. (A) Normal Vero cells; (B) Vero cells infected fecal specimen becomes rounded and detached from flask. C-D 20–30 nm virus particles were observed by electron microscope (EM) in the tissue culture infected with fecal specimen and spleen specimen (passage 5), indicated by white arrow. The scale bar is 100 nm.

To determine the potential agents involved in this outbreak, the fecal and spleen samples from 4 sick/sacrificed goats were processed, respectively, to inoculate the Vero cells for virus isolation. After incubation with the inoculums, Vero cells showed cytopathic effects (CPE) as early as 8–10 h. Compared with normal Vero cells (Fig 1A), infected cells initially became rounded before the majority of the cells detached off the flask 24–48 h post infection (PI) (Fig 1B).

To rule out the possibility that the CPEs were caused by the potential toxic effect of the inoculums, the infected cells were frozen, thawed and used as inoculums to infect cells at least 5 passages. Similar CPEs were also observed in Vero cells after they were infected with viruses from different passages (not shown), suggesting the CPEs were indeed caused by viral pathogens. Further examination of infected cell cultures using electron microscope (EM) revealed 20–30 nm virus particles in the samples (Fig 1C and 1D). The viruses were isolated using Vero cells or detected using RT-PCR from three out of four fecal samples and two of four spleen samples collected respectively from 4 goats examined (Table 3), indicating that the viruses are likely associated with the outbreak. The isolated virus particles were designated as CEV-JL14.

**Table 3:** Virus isolation or detection from samples of spleen and feces in goats.

Goat No	Clinical signs	Feces	Feces	Spleen	Spleen
		isolation	EM	Isolation	EM
14	Diarrhea, pneumonia	+	+	+	+
24	Diarrhea, pneumonia	+	+	-	-
42	diarrhea	+	+	-	-
57	Watery diarrhea	+	+	+	+

+: virus was isolated and identified; -: no virus was isolated; EM: electron microscope

### Virus titration and physiochemical properties

To determine the virus infectivity, TCID_50_ was determined following the protocol as previously described[24]. Serially diluted viruses were inoculated to quadruplicate samples in 96-well microplate. After 48–72 h, the wells showing CPE were counted. The TCID_50_ for CEV-JL14 strain in 5^th^ passage from three repeats was shown to be 10^7.4^pfu/0.1ml. Characterization of physicochemical properties of CEV-JL14 showed that it was resistant to treatment in low pH (3.0). Treatment of CEV-JL14 with chloroform/ ether had no significant effects on its infectivity, suggesting it is a non-enveloped virus.

### CEV-JL14 is a caprine enterovirus

To define the viral pathogen, PCRs were performed to identify the potential viruses using primers for parvovirus, enterovirus and foot and mouth disease virus (FMDV) since those viruses had the similar sizes of 22–30 nm. No fragments were obtained using primers for parvovirus and FMDV after different amplification conditions were tried, ruling out the possibility that those viruses are associated with the disease. However, fragment was amplified using primers selected based on the genome sequence comparison for EV-E, EV-F and EV-G (Table 1), suggesting that the virus is likely an enterovirus. Sequencing the PCR-amplified fragment (partial 5’-UTR) revealed that the fragment indeed contained a nucleotide sequence of enterovirus. This partial 5’-UTR fragment shared 86% sequence identity with the corresponding region of the ovine enterovirus strain TB4-OEV, confirming that the isolated virus is enterovirus.

### The complete genome sequence of CEV-JL14

To unveil the genome sequence of CEV-JL14, the primers listed in Table 1 were designed based on the nucleotide sequence of TB4-OEV, and used to amplify the viral complete genome. Complete genome sequence of CEV-JL14 was obtained by joining the nucleotide sequence of several overlapping PCR amplified fragments. Sequence analysis showed that the complete genome sequence of CEV-JL14 consists of 7461 nucleotides, with a typical picornavirus genome organization including a 5’-UTR of 821 nucleotides, a large single ORF between nucleotides 822 and 7340, and a 3’-UTR of 119 nucleotides consisting of 51 adenines (poly A) tail. The ORF encodes a polyprotein of 2172 amino acids with a predicted molecular weight of 241kD. The complete nucleotide sequence of CEV-JL14 was deposited in GenBank with accession number KU297674.

### CEV-JL14 had the highest sequence identity to ovine enterovirus TB4-OEV

Since CEV-JL14 was the first caprine enterovirus with its complete genome sequence revealed, it is natural to determine its complete sequence homology with other enteroviruses. As shown in Table 4, CEV-JL14 had 77.1% complete sequence identity with ovine enterovirus TB4-OEV, 69.5–72.1% with EV-G, and 62.8–68.3% with EV-E and EV-F, and 56.3–57.3% with EV-A-D. The deduced amino acid sequence identity of CEV-JL14 was 89.3% with TB4-OEV, 79.6–82.2% with EV-G and 64.8–65.6% with EV-E and EV-F, and 55.4–57.2% with EV-A-D. Those results indicate CEV-JL14 had a closer relationship with TB4-OEV and EV-G than other enterovirus species including EV A, EV B, EV C, EV D, EV-E and EV-F.

**Table 4:** Sequence identities of CEV-JL14 with known representative enterovirus species (A, B, C, D, E, F, G, H, J).

Isolates	Species	5’UTR	COM		VP1		P1
		%(nt)	%(nt)	%(aa)	%(nt)	%(aa)	%(aa)
TB4-OEV-2009-HUN	?	82.3	77.1	89.3	64.7	68.8	81.1
PEV-B-KOR	G	52.9	70.2	79.8	30.9	60.4	69.7
PEV-CH-AH-F1	G	52.3	null	null	50.3	57.0	null
PEV-K23-2008-HUN	G	52.1	69.6	81.2	59.5	60.0	72.3
PEV10-LP54	G	56.8	72.1	82.2	65.3	68.0	74,7
PEV-WBD-2011-HUN	G	51.8	69.5	79.6	60.3	62.6	71.0
BEV-261	F	78.8	63.7	65.3	53.5	52.5	61.9
BEV-3A	F	80.3	64.3	65.6	52.4	51.3	62.0
BEV-BHM26	F	78.4	63.5	65.5	52.8	53.3	61.4
BEV-BJ001	F	69.2	64.1	65.6	53.3	51.0	61.7
BEV-PS 87	F	78.5	68.3	64.8	52.4	50.6	61.0
BEV-HY12	E	72.9	62.8	65.3	51.8	50.0	62.5
BEV-LC-R4	E	75.7	63.6	65.3	51.9	48.5	62.0
BEV-PA12-24791	E	73.7	63.5	65.2	51.9	48.5	62.2
BEV-PS 83	E	74.5	63.0	64.9	52.3	50.0	62.1
BEV-SL305	E	74.0	63.4	65.5	52.3	50.4	63.1
CBV3-18219-02	B	54.2	56.8	56.9	44.4	39.0	48.9
EV11-18744-02	B	53.5	57.3	56.7	43.8	38.8	47.8
EV7-15936-01	B	54.5	57.0	56.6	45.0	37.7	47.9
EV30-8477-98	B	53.4	57.1	56.6	46.1	38.8	50.2
S0098b/CA16/2013/CHN	A	54.6	57.0	57.0	51.5	45.2	52.3
S0102b/EV71/2013/CHN	A	54.8	57.1	57.2	49.3	40.7	52.9
Sev-nj1	J	57.3	58.9	59.2	51.1	43.2	52.9
1631	J	55.5	58.7	60.5	51.4	41.2	54.6
EV-D68	D	56.3	56.8	55.7	46.7	41.3	51.5
Beijing-R0132	D	57.0	56.8	55.6	46.7	41.3	51.2
US/KY/14-18953	D	56.8	56.8	55.7	47.2	40.5	51.1
Brunenders	C	55.9	56.7	55.9	45.0	39.5	46.6
V2-Tol.1	C	53.9	56.3	55.4	45.7	38.5	46.1
V3-Tul.7	C	54.6	56.7	55.4	44.4	37.0	45.7

### CEV-JL14 had a higher similar 5’-UTR secondary structure to BHM26 than other *Enterovirus* species

To determine the secondary structure feature of CEV-JL14, its 5’-UTR sequence was analyzed using RNAfold software as described previously [27]. The predicted secondary structure of CEV-JL14 was compared with TB4-OEV and representative enterovirus from different *Enterovirus* species including EV-E, EV-F, EV-G and EV-A. As shown in S1 Fig, the 5’-UTR secondary structures of CEV-JL14 consisted of 7 stem-loop structures that had difference to those of TB4-OEV, PEV10 LP54 (EV-G), EV71 (EV-A), HY12 (EV-E), and BHM26 (EV-F) where it shared more similarity to BHM26. Those results demonstrated the divergence of 5’-UTR structure among CEV-JL14 and other *Enterovirus* species (EV-E, EV-F, EV-G and EV-A). Similar to the above results, the mountain plot of MEF structure, the thermodynamic ensemble RNA structure, and centroid structure of CEV-JL14 were also different from those of TB4-OEV, PEV10 LP54, EV71, HY12, and BHM26 strains (S2 Fig).

### CEV-JL14 belongs to a novel species within the genus *Enterovirus*

**Fig 2:**
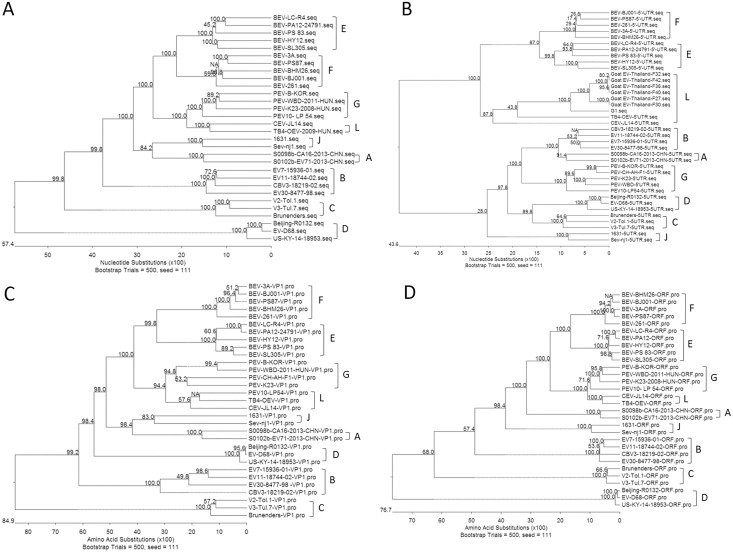
CEV-JL14 belongs to a novel species within the genus. Phylogenetic analysis of CEV-JL14 with representative EV-E, EV-F, EV-G and strains of other enterovirus species. (A) The phylogenetic tree generated by analyzing the complete genome sequence of CEV-JL14 with the representative EV-E, EV-F, EV-G and other enterovirus species. (B-C) Phylogenetic analyses of CEV-JL14 with the representative EV-E, EV-F, EV-G and strains of other species performed with the 5’-UTR, VP1 amino acid sequences by neighboring-joining methods. (D) Phylogenetic analysis of polyprotein sequence of CEV-JL14 with the representative EV-E, EV-F, EV-G and strains of other species. (E) Phylogenetic analysis of P1 of CEV-JL14 with the representative EV-E, EV-F, EV-G and strains of other species. The CEV-JL14 marked as solid triangle, together with TB4-OEV or other ovine/caprine enterovirus was clustered to a novel clade enterovirus L (EV-L). All the phylogenetic analyses above were performed using neighbor-joining method with a bootstrap value of 500.

The criteria for classification of the unknown virus in the genus of *Enterovirus* is polythetic and normally based on the sequence variation of the 5’-UTR and the capsid structural genes[20, 23]. To determine the phylogenetic status of CEV-JL14, the complete nucleotide sequences of CEV-JL14 and its encoded individual gene were aligned with the corresponding region of known representative enteroviruses. As shown in Fig 2A, alignment analysis of CEV-JL14 complete genome sequence with representative enterovirus species clustered the enteroviruses to 9 clades including EV-A, EV-B, EV-C, EV-D, EV-E, EV-F, EV-G, EV-J and a new clade that only consists of CEV-JL14 and TB4-OEV. Alignment analysis with 5’-UTR sequences also demarcates the enteroviruses into 9 clades where the CEV-JL14 was neither clustered to the clade of EV-E and EV-F, nor to the clade of EV-G and EV-A-D. However, it is surprising to note that CEV-JL14, together with several partial goat enteroviruses and TB4-OEV, was clustered to a distinct clade from those of EV-E, EV-F and EV-G, EV-A, EV-B, EV-C, and EV-D, suggesting the viruses in the distinct clade is the novel species within the genus of *Enterovirus*. We named the enterovirus in this clade (caprine enterovirus and ovine enterovirus) as *Enterovirus* species L (EV-L).

**Fig 3:**
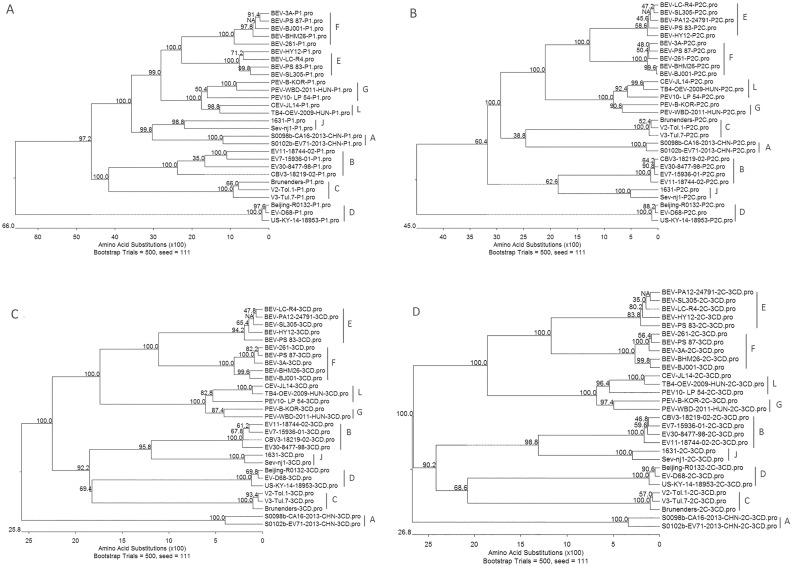
Phylogenetic analysis of CEV-JL14 with representative enteroviruses from other *Enterovirus* species. Phylogenetic analysis of P1 (A), P2C (B), P3CD (C) and P2C+P3CD (D) of CEV-JL14 with the representative EV-E, EV-F, EV-G and strains of other species. The CEV-JL14 marked as solid triangle, together with TB4-OEV, CEV-JL14 was clustered to a novel clade enterovirus L (EV-L). All the phylogenetic analyses above were performed using neighbor-joining method with a bootstrap value of 500.

Similar patterns were also observed when the VP1 amino acid sequence (Fig 2C), the polyprotein sequences (Fig 2D), P1, P2C, and P3CD amino acid sequence (Fig 3) were applied for alignment analyses. The VP1 nucleotide sequence identity of CEV-JL14 was 64.7% to TB4-OEV, 51.8–53.5% to EV-E and EV-F, 30.9%-65.3% to EV-G, and 43.8–51.5% to EV-A-D. The P1 amino acid sequence identity of CEV-JL14 was 81.1% to TB4-OEV, 61.0–63.1% to EV-E and EV-F, 69.7–74.7% to EV-G, and 45.7–52.9% to EV-A-D (Table 4). Analysis of the P1 amino acid sequence revealed higher amino acid sequence identity of P1 intraspecies for EV-E, EV-F and EV-G, respectively, where it was 86.2–96.7% among viruses within EV-E, 84.5–95.5% within EV-F, and 73.4–85% within EV-G, respectively. Those results showed that P1 sequence identity was much higher among viruses in the same species, and demarcated the CEV-JL14 and TB4-OEV from EV-E, EV-F, EV-G and other enterovirus species (EV-A-D).

To confirm the above results, the percentage of the G+C content of CEV-JL14, TB4-OEV, and representative EV-G were calculated. As shown in S1 Table, TB4-OEV had 48.75% G+C content, which had 2.5% percentage difference of G+C content over those viruses in the EV-G (45.45–46.25%), suggesting TB4-OEV should not listed as member of EV-G according to the criteria that G+C contents should be less than 2.5% difference if the enteroviruses were within the same species. The G+C content for CEV-JL14 is 48.26%, which also had 2.5% G+C content difference to PEV10 LP54 and PEV-B-KOR strain in EV-G. Those results, contradicting to the classification criteria, suggest that CEV-JL14 and TB4-OEV should be not grouped in EV-G although TB4-OEV was originally listed as member of EV-G. Instead, they should be grouped as a new enterovirus species.

To further ensure the above claim, the potential proteolytic sites for CEV-JL14, TB4-OEV, and viruses in EV-G were comparatively analyzed. As shown in S2 Table, 3 out of 10 potential proteolytic cleavage sites including VP4/VP2, P2B/P2C, P3A/P3B shared the same amino acid sequence for CEV-JL14, TB4-OEV and viruses in EV-G, while 6 out of 10 potential proteolytic processing sites shared the same proteolytic sequence between CEV-JL14 and TB4-OEV. Those results demonstrated the proteolytic sites are highly conserved between TB4-OEV and CEV-JL14, and highly variable among CEV-JL14, TB4-OEV and EV-G, indicating that CEV-JL14 and TB4-OEV were different from EV-G viruses.

Taken together, the above results demarcate CEV-JL14 from EV-E, EV-F, and EV-G and support our claim that CEV-JL14, together with TB4-OEV, should be grouped as new *Enterovirus* species, designated as EV-L within the genus of *Enterovirus*.

Sequence comparison between CEV-JL14 and TB4-OEV revealed 82.3% sequence identity for 5’-UTR, and 64.7% for VP1, respectively (Table 4). Those results suggest a high nucleotide sequence divergence even between sheep and goat enterovirus and indicate a different subgenotype for ovine enterovirus and caprine enterovirus, respectively.

## Discussion

In this study, we have isolated and characterized a novel enterovirus from goats characterized by severe diarrhea with a high morbidity and mortality. Sequence analysis clearly distinguishes the CEV-JL14 from the EV-E, EV-F, EV-G, and other known enterovirus species based on the current criteria for enterovirus classification. Our phylogenetic analysis showed that CEV-JL14 clustered to neither EV-E, EV-F, EV-G nor other enterovirus species, it is clustered to a novel distinct clade together with TB4-OEV and Caprine enterovirus G1 from Japan and recently reported enterovirus in goats from Thailand. Pursuant to current criteria for classification of enterovirus species, where a range from 50 to 55% for heterologous species, 70 to 85% for heterologous serotypes/homologous species and greater than 90% for homologous serotypes[23], we proposed that CEV-JL14, together with TB4-OEV and other ovine/caprine enterovirus strains, was classified to a novel enterovirus species L (EV-L). This proposal is based on our following findings: 1) the nucleotide sequence identity of VP1 for CEV-JL14 was only 51.8–53.5% to EV-E and EV-F, 30.9%-65.3% to EV-G, and 43.8–51.5% to EV-A-D, which is well-matched with the criteria that 50–55% of nucleotide sequence belongs to heterogeneous species; 2) the deduced VP1 amino acid sequence were only 48.5%-53.3% to EV-E and EV-F, 57.0%-68.0% to EV-G, and 37.0–45.2% to EV-A-D, which is also consistent with the enterovirus species criteria; 3) CEV-JL14, together with TB4-OEV, was clustered to a distinct clade from EV-E, EV-F, EV-G, and EV-A-D based on the 5’-UTR sequence analysis and their secondary structure prediction. 4) The P1 amino acid sequence identity of CEV-JL14 was 81.1% to TB4-OEV, 61.0–63.1% to EV-E and EV-F, 69.7–74.7% to EV-G, and 45.7–52.9% to EV-A-D. 5) the percentage difference of G+C content for CEV-JL14 and TB4-OEV to EV-G is more than 2.5%, suggesting that CEV-JL14 and TB4-OEV should not listed as member of EV-G. 6) The proteolytic cleavage sites between CEV-JL14 and TB4-OEV is highly conserved, while they are highly variable between CEV-JL14/TB4-OEV and EV-G. Those results support our claim that CEV-JL14, together with other ovine/caprine enterovirus strains, ought to be considered as new species in the genus of *Enterovirus*. Although cladograms variations were revealed employing different capsid genes for the analysis, similar cladogram patterns were observed even under circumstance of the lack of enough virus isolates. With more and more caprine or ovine enterovirus sequences revealed, the clade will become much clearer.

TB4-OEV was the first ovine enterovirus isolated with its complete genome sequence revealed, and analysis of TB4-OEV sequence grouped this virus to EV-G, a *Enterovirus* species that caused swine infection mainly[5]. Our phylogenetic analysis clustered the TB4-OEV and the caprine enterovirus CEV-JL14 to a novel enterovirus named enterovirus L (EV-L) within the genus of *Enterovirus*. The discrepancy between our analysis and previous analysis is likely due to either lack of ovine/caprine enterovirus sequences or the majority of goat enterovirus sequences was just a partial 5’-UTR sequences[5, 19]. To our knowledge, this is the first enterovirus isolate from goats in China and first caprine enterovirus with its complete genomic sequence revealed, which will enrich the current understanding of enterovirus variation and evolution.

Although enteroviruses have been demonstrated to be the leading agents related to human respiratory, digestive and neurological disorder including the poliomyelitis, coxsackievirus infection, echovirus infection and hand, food, mouth diseases (HFD), the enterovirus infection in animals remains largely unexplored. The frequent isolation or detection of a large amount of enterovirus particles from animals characterized with respiratory and digestive diseases with high morbidity and mortality should arouse attention to this emerging infection. The isolation of enterovirus from goats with severe diarrhea and detection of the virus antigens in the tissue of the diseased goats suggest that the enterovirus likely play a crucial role in the outbreak characterized in this study. Future investigation will focus on unveiling the mechanism underlying the involvement of enteroviruses in the severe respiratory and digestive diseases in animals. The finding that sequence divergence between the caprine enterovirus and ovine enterovirus indicates that enteroviruses co-evolved with the host species, which is also an interesting topic for future investigation.

## Supporting information

S1 Fig The 5’-UTR secondary structure of CEV-JL14. The 5’-UTR secondary structure of CEV-JL14 was predicted using minimal free energy with the methods described previously [27] (A) and compared with that of TB4-OEV (B), PEV10 LP54 (C), EV71 (D), HY12 (E) and BHM26 (F).
(TIF)

S2 Fig The mountain plot of 5’-UTR secondary structure for CEV-JL14. The mountain plot comparison of CEV-JL14 with that of TB4-OEV (B), PEV10 LP54 (C), EV71 (D), HY12 (E) and BHM26 (F).
(TIF)

S1 Table G+C contents among the different *Enterovirus* species within the genus of *Enterovirus* of the *Picornaviridae*. (DOCX)

S2 Table Proteolytic processing sites of majority of enterovirus G strain, TB4-OEV and CEV-JL14. (DOCX)
