# Supplementary figures and images for: A novel enterovirus species identified from severe diarrheal goats
Source: PLoS One. 2017 Apr 4;12(4):e0174600. doi: 10.1371/journal.pone.0174600 (PMC5380325; doi:10.1371/journal.pone.0174600)

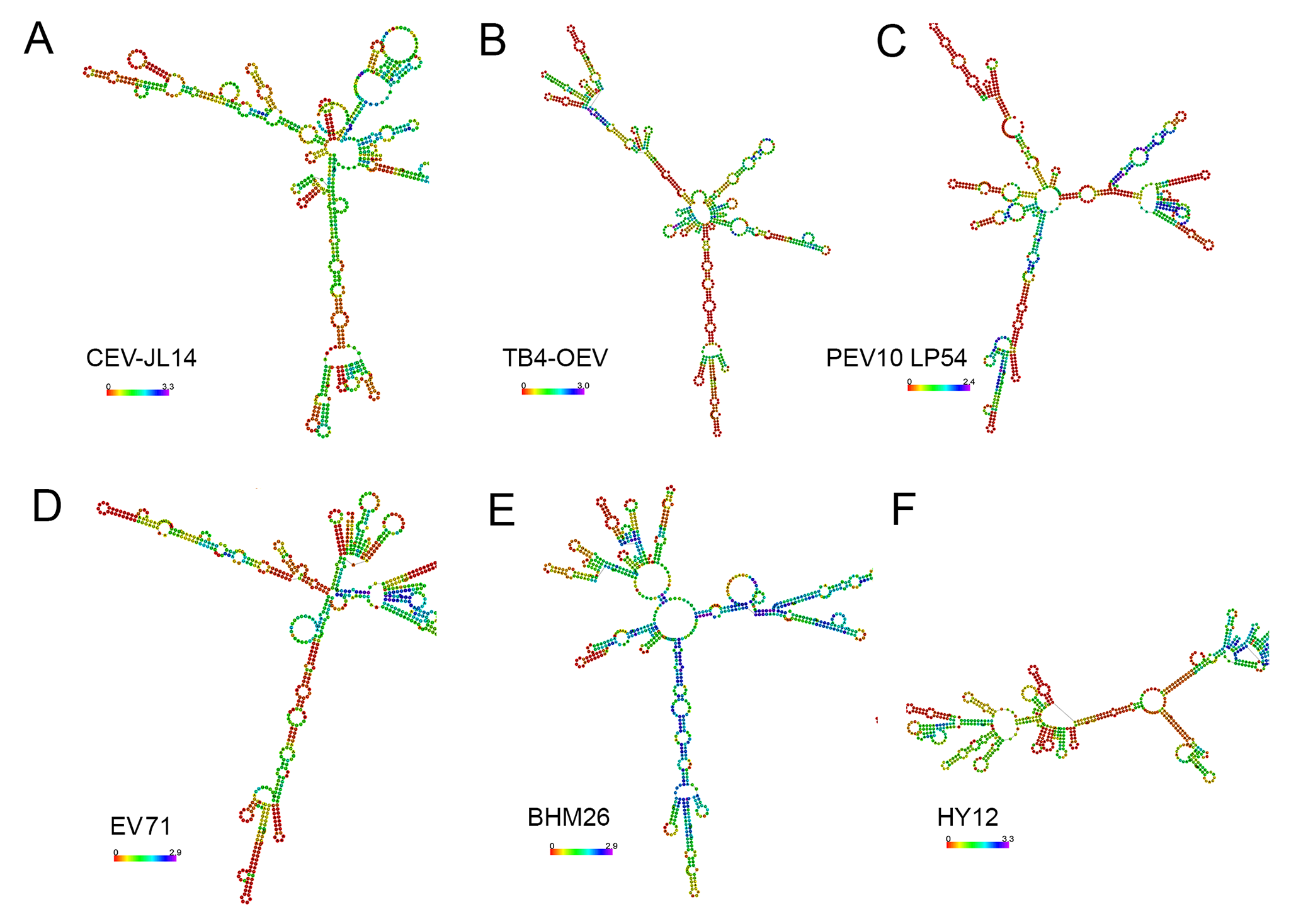

Supplement: S1 Fig — The 5’-UTR secondary structure of CEV-JL14 was predicted using minimal free energy with the methods described previously [27] (A) and compared with that of TB4-OEV (B), PEV10 LP54 (C), EV71 (D), HY12 (E) and BHM26 (F). (TIF) [file pone.0174600.s001.tif]

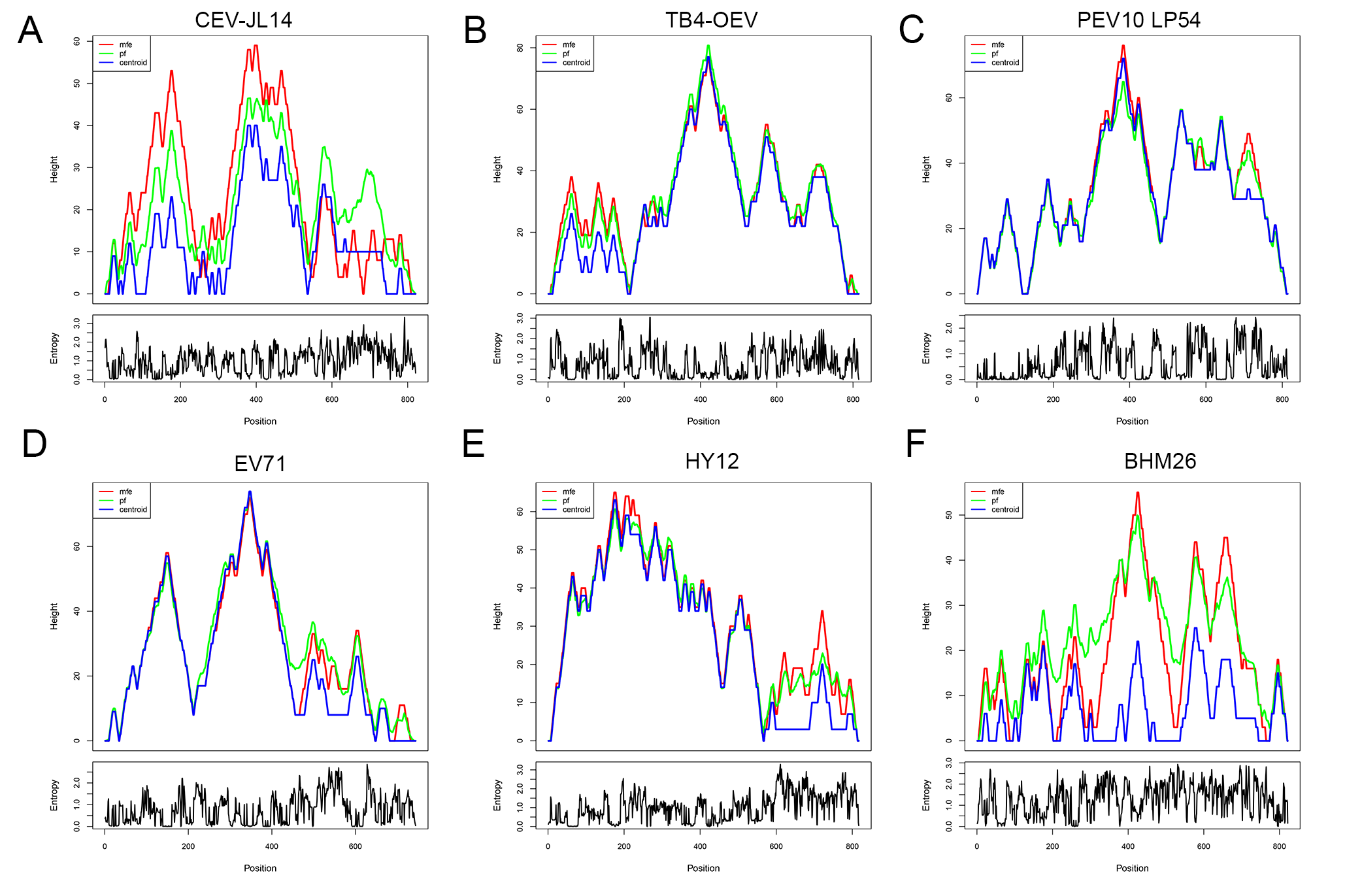

Supplement: S2 Fig — The mountain plot comparison of CEV-JL14 with that of TB4-OEV (B), PEV10 LP54 (C), EV71 (D), HY12 (E) and BHM26 (F). (TIF) [file pone.0174600.s002.tif]
