# Supplementary material for: A novel enterovirus species identified from severe diarrheal goats
Source: PLoS One. 2017 Apr 4;12(4):e0174600. doi: 10.1371/journal.pone.0174600 (PMC5380325; doi:10.1371/journal.pone.0174600)
Supplement: S1 Table — (DOCX) [file pone.0174600.s003.docx]

Table S1 G+C contents among the different *Enterovirus* species within the genus of *Enterovirus* of the *Picornaviridae*

| Name of Strains | G+C content | designated Species |
| --- | --- | --- |
| CEV-JL14 | 48.26 | L |
| TB4-OEV-2009-HUN | 48.76 | G(?) |
| PEV10-LP54 | 45.45 | G |
| PEV-B-KOR | 45.72 | G |
| PEV-K23-2008-HUN | 46.25 | G |
| PEV-WBD-2011-HUN | 45.92 | G |
| BEV-261 | 50.52 | F |
| BEV-3A | 50.99 | F |
| BEV-BHM26 | 50.72 | F |
| BEV-BJ001 | 50.77 | F |
| BEV-PS 87 | 51.32 | F |
| BEV-HY12 | 48.55 | E |
| BEV-LC-R4 | 50.53 | E |
| BEV-PA12-24791 | 48.97 | E |
| BEV-PS 83 | 50.49 | E |
| BEV-SL305 | 49.78 | E |
| CBV3-18219-02 | 47.83 | B |
| EV11-18744-02 | 47.74 | B |
| EV7-15936-01 | 47.47 | B |
| EV30-8477-98 | 47.71 | B |
| S0098b/CA16/2013/CHN | 47.10 | A |
| S0102b/EV71/2013/CHN | 47.93 | A |
| Sev-nj1 | 47.08 | J |
| 1631 | 45.29 | J |
| EV-D68 | 41.41 | D |
| Beijing-R0132 | 41.76 | D |
| US/KY/14-18953 | 41.07 | D |
| Brunenders | 46.48 | C |
| V2-Tol.1 | 46.12 | C |
| V3-Tul.7 | 46.49 | C |
