# Supplementary material for: A novel enterovirus species identified from severe diarrheal goats
Source: PLoS One. 2017 Apr 4;12(4):e0174600. doi: 10.1371/journal.pone.0174600 (PMC5380325; doi:10.1371/journal.pone.0174600)
Supplement: S2 Table — (DOCX) [file pone.0174600.s004.docx]

Table S2 Proteolytic processing sites of majority of enterovirus G strain, TB4-OEV and CEV-JL14

| Proteolytic sites | Enterovirus G | TB4-OEV | CEV-JL14 |
| --- | --- | --- | --- |
| VP4/VP2 | VPL/KSP | VPL/KSP | VPL/KSP |
| VP2/VP3 | AIA/QGI | AIV/QGI | AIT/QLP |
| VP3/VP1 | CLM/AAQ | CLV/AAQ | CLV/AAQ |
| VP1/2A | VTN/TGA | ITT/TGA | NIT/TGA |
| 2A/2B | AME/QGL | VME/QGI | AME/QGI |
| 2B/2C | VEK/QGD | VEK/QGD | VEK/QGD |
| 2C/3A | LFG/GPP | LFQ/GPP | LFQ/GPP |
| 3A/3B | AGY/QGP | AGY/QGP | AGY/QGP |
| 3B/3C | KAQ/GPL | RAQ/GPL | KAQ/GPL |
| 3C/3D | KPQ/GQI | KEQ/GKI | KEQ/GKI |
